# Supersensitive and robust disease monitoring in oropharyngeal cancer patients by circulating tumor HPV-DNA sequencing (ctHPV-DNAseq)

**DOI:** 10.1016/j.tranon.2026.102744

**Published:** 2026-04-01

**Authors:** A.S. Pierik, J.B. Poell, A. Brink, M. Stigter-van Walsum, F. Jansen, R. de Bree, J. Hardillo, J.A. Langendijk, R.P. Takes, F. Lamers, I.M. Verdonck-de Leeuw, J.J. Hendrickx, S.A. Koppes, F. Rosing, T. Waterboer, C.R. Leemans, R.H. Brakenhoff

**Affiliations:** a Amsterdam UMC location Vrije Universiteit Amsterdam, Otolaryngology/Head and Neck Surgery, De Boelelaan 1117, Amsterdam, The Netherlands; b Cancer Center Amsterdam, Imaging and Biomarkers, Amsterdam, The Netherlands; c Cancer Center Amsterdam, Treatment and Quality of Life, Amsterdam, The Netherlands; d Department of Head and Neck Surgical Oncology, University Medical Center Utrecht, Utrecht, The Netherlands; e Department of Otorhinolaryngology, Erasmus MC Cancer Institute, University Medical Center, Rotterdam, The Netherlands; f Department of Radiation Oncology, University Medical Center Groningen, Groningen, The Netherlands; g Department of Otorhinolaryngology-Head and Neck Surgery, Radboud University Medical Center, Nijmegen, The Netherlands; h Amsterdam UMC location Vrije Universiteit Amsterdam, Psychiatry, Amsterdam, The Netherlands; i Amsterdam Public Health, Mental Health, Amsterdam, The Netherlands; j Vrije Universiteit Amsterdam, Clinical, Neuro and Developmental Psychology, Van der Boechorststraat 7-9, Amsterdam, The Netherlands.; k Department of Pathology, Erasmus MC Cancer Institute, University Medical Center, Rotterdam, The Netherlands; l Division of Infections and Cancer Epidemiology, German Cancer Research Center (DKFZ)

**Keywords:** HNSCC, liquid biopsy, Recurrence, HPV-positive, DNA sequencing, Circulating tumor DNA, ctDNA

## Abstract

**Background:**

Post-treatment disease monitoring of HPV-positive oropharyngeal squamous cell carcinoma (OPSCC) is challenging. Liquid biopsies could improve disease monitoring, but the variety in methods hampers clinical implementation. In this study, target-enrichment sequencing to detect circulating tumor HPV DNA (ctHPV-DNA) was applied in liquid biopsies of HPV-positive OPSCC patients, and robust statistical readouts determined. Next, it was investigated whether longitudinal plasma monitoring could accurately diagnose residual and recurrent disease.

**Methods:**

The target-enrichment panel included 29 cancer genes and high-risk HPV genomes. The assay was tested on plasma from 30 non-cancer controls and 33 patients with HPV-positive tumors, 15 of whom had residual or recurrent disease, and 18 who remained disease-free. Samples were analyzed from baseline to 24 months after treatment.

**Results:**

By determining and applying robust statistical cut-off values, ctHPV-DNA could be detected in plasma of all patients with HPV-positive OPSCC at baseline, and was absent in plasma of all non-cancer controls. In OPSCC patients who remained disease-free, post-treatment plasma samples were negative for ctHPV-DNA. In contrast, ctHPV-DNA was detected in plasma of all OPSCC patients with recurrent disease to a year before clinical diagnosis. Cases suspect for residual disease in the neck, but with a necrotic metastasis without vital tumor after resection, tested correctly negative for ctHPV-DNA in plasma.

**Conclusions:**

Target-enrichment sequencing of plasma shows 100% accurate detection of ctHPV-DNA at baseline. Longitudinal monitoring enables early recurrence detection and correct diagnosis of non-vital residual disease. The data indicate that liquid biopsy could improve post-treatment follow-up in HPV-positive OPSCC patients.

## Highlights

• Detection of ctHPV-DNA by target-enrichment DNA sequencing including selected human cancer genes (ctHPV-DNAseq) allows determination of robust statistical cut-offs and provides a variety of quality controls that will facilitate clinical implementation of liquid biopsy disease monitoring.
• Detection of ctHPV-DNA by target-enrichment DNA sequencing in plasma of HPV-positive oropharyngeal cancer patients and non-cancer controls is 100% accurate at baseline.
• Detection of ctHPV-DNA by target-enrichment DNA sequencing demonstrates a 10-100x higher analytical sensitivity than digital PCR.
• Plasma ctHPV-DNA detection by target-enrichment DNA sequencing provides an extremely valuable tool for the detection of recurrent and residual oropharyngeal cancer, outperforming standard clinical practice.

## Introduction

Head and neck squamous cell carcinoma (HNSCC) arises from the mucosal lining of the upper aerodigestive tract as the sixth most common cancer worldwide, and accounts for approximately 5% of all malignancies [1,2]. Several risk factors have been implicated in the development of HNSCC, including tobacco use, alcohol consumption, betel nut chewing, and human papillomavirus (HPV) infection [3]. HPV-positive tumors typically occur as squamous cell carcinomas in the oropharynx (OPSCC), and are considered a distinct clinical entity with unique clinical and molecular characteristics. These tumors are generally treated with definitive radiotherapy, chemoradiotherapy, or primary surgery such as transoral robotic surgery (TORS) depending on the disease stage. HPV-positive OPSCC accounts for a significant proportion of all HNSCC cases in Western countries, and the incidence of HPV-positive OPSCC has been rising steadily [[4], [5], [6], [7], [8]].

The clinical management of patients with HPV-positive OPSCC during follow-up has limitations. Current disease monitoring according to WHO guidelines consists of treatment response evaluation by MRI and/or PET-CT three to four months after treatment completion, and clinical follow-up visits every 6-8 weeks in the first year, every 8-10 weeks in the second year and once every half year in years 3-5. When imaging for response evaluation is performed, residual disease is detected in 10-20% either at the local site or regionally in the neck. This requires a subsequent diagnostic workup by taking biopsies to diagnose vital tumor at the primary site, generally under general anesthesia. For the neck ultrasound-guided guided fine needle aspiration cytology is applied [9]. As samples are taken from post-treatment tissue areas, histo- and cytopathological examination is often not conclusive. Consequently imaging is repeated or a surgical procedure planned, and the excised residual tumor may emerge as a necrotic tumor mass without any vital tumor cells. Besides the diagnostic issues of residual disease, locoregional recurrences of HPV-positive HNSCC tumors may still occur in 10-15% of cases, necessitating an adequate clinical follow-up management.

Hence, current disease monitoring of treated HPV-positive oropharyngeal cancer patients has limited sensitivity and specificity, leading to incorrect diagnosis and even unnecessary treatments of residual disease with non-vital tumor in the surgical specimen. In addition detection of locoregional recurrences is generally late. These findings highlight the need for better follow-up surveillance strategies for HPV-positive OPSCC patients.

Circulating tumor human papillomavirus (ctHPV) DNA has emerged as a promising biomarker for the detection of HPV-positive OPSCC. CtHPV DNA can be detected in the blood, saliva and oral rinse of HPV-positive HNSCC patients and has been shown to be a sensitive and specific marker for disease recurrence [10]. Different methods for detection have been used, including quantitative PCR [11], digital droplet PCR (ddPCR) [12], and low coverage whole genome sequencing [13]. Target-enrichment approaches have previously been used to detect rare somatic variants in cancer genes [14], and these methods have been applied for detection of HPV as well [15].

A potential problem with the various methods is the definitive readout as well as the determination of the cut-off values, varying from the number of HPV reads combined with the coverage of the HPV genome [16], or HPV copies per ml [15]. Clinical implementation will be favored by consistent positive or negative calls based on robust statistical cut-off values. In addition, a sample can only be indicated as negative when a certain minimum sequence coverage has been reached. The aim of this study is to investigate application of statistical cut-off values for calling a sample positive or negative for ctHPV-DNA sequencing, to test the added value of the target-enrichment, and to demonstrate whether longitudinal monitoring of ctHPV-DNA in plasma and oral rinse during post-treatment follow-up with this assay could accurately diagnose residual disease or predict recurrent disease in HPV-positive OPSCC patients. Finally we compare the analytical sensitivity of DNA sequencing with dPCR.

## Patients and methods

### Patients

Patient data and samples were collected from HNSCC patients and non-cancer controls (caregivers) in the NET-QUBIC study (2014-2018) with 739 HNSCC patients and 262 controls, and the HN-Detect study (2021-2023) with 41 patients. The HN-DETECT study is an observational prospective cohort study collecting plasma samples and oral rinses during post-treatment follow-up to detect residual or recurrent disease, Dutch CCMO trial register number NL72940.029.20. Patients with HPV-positive OPSCC and a complete follow-up of 3 years were selected from the HN-DETECT study. Samples were taken at baseline and follow-up visits (6, 12, and 24 months after treatment for NET-QUBIC, and every follow-up visit for HN-Detect. All OPSCC tumors from both studies were tested at baseline for HPV by p16 immunohistochemistry followed by HPV DNA PCR on the p16-immunopositive cases [17,18]. In four cases only p16-immunostating was performed. Both studies were approved by the Institutional Review Board, and all participants signed informed consent. Only patients with p16 and HPV-DNA positive tumors (n=29) or a positive baseline plasma sample (n=4) were included. Recurrent and residual disease patients were matched with disease-free patients for stage and treatment, and non-cancer controls were matched with patients for age, smoking, and alcohol use.

### Sequencing and data analysis

Sequencing and data analysis are described in detail in the Supplementary methods. In brief, DNA was isolated by Qiagen columns and prepared for sequencing using the KAPA HyperPrep kit (Roche Diagnostics Nederland BV, Almere, The Netherlands). The gene and HPV panel enriched for sequencing is indicated in Supplementary table 1. Libraries were pooled and sequenced on an Illumina Novaseq (Illumina Nederland BV, Eindhoven, the Netherlands) by 250 bp paired-end runs. Sequencing data were mapped to a combined human-HPV reference genome (hg19) with all 15 high-risk HPV types added as separate chromosomes, and reads mapping to either human genome or HPV were enumerated.

### Statistical analysis

Determination of the cut-off values are explained in detail in the Supplementary methods. In short, the value of HPV reads per human genome reads was determined. The log-transformed HPV per human genome values of the plasma samples at baseline of HPV-positive patients were used to calculate the mean and determine the bounds of the 99.7 confidence interval (3x the standard deviation) for the distribution of positive samples. The lower bound of the confidence interval was used to call a sample positive or negative, above or below the threshold respectively. The calculation of the minimally number of sequenced human genome reads to call a sample negative is explained in the Supplementary methods.

Plasma DNA yield between OPSCC patients and non-cancer controls was compared with an unpaired t-test. Fragment lengths of human reads versus HPV reads within one sample were compared with a paired t-test. Sensitivity for HPV detection between lcWGS and HPV-enriched sequencing was analyzed by McNemar’s Chi-squared test. Disease-free survival was analyzed by the Kaplan-Meier method and significance between the curves determined by the logrank test. Statistical analyses were performed in R.

### dPCR vs target enrichment sequencing

To compare the sensitivity of target enrichment sequencing and dPCR, we assessed the limit of detection (LOD) for each method using a serial dilution of plasma DNA from an HPV-positive OPSCC patient in plasma DNA of an HPV-negative oral cancer patient. This mimics most closely the real situation of circulating plasma DNA that is fragmented. From the HN-DETECT study, two plasma samples at baseline were obtained from an HPV-positive patient, and several longitudinal samples were pooled from an HPV-negative oral cancer patient. DNA was extracted from the plasma samples according to the procedure outlined above and in the Supplementary methods. To generate a sufficient volume for the serial dilution, 100 µL of AVE buffer (Qiagen DNA isolation kit) was added to the pooled DNA sample of the HPV-negative oral cancer patient. The dilution series was prepared by adding 6 µL of HPV-positive plasma DNA to 54 µL of control HPV-negative plasma DNA, and this mixture was further serially diluted until a final dilution of 1/100,000 was reached consisting of five serial dilution steps. The serially diluted samples were subsequently split into two vials, each containing 25 µL of the sample. These samples were used for dPCR or target enrichment DNA sequencing (ctHPV-DNAseq).

Target enrichment DNA sequencing was performed as described above and dPCR was described in Rosing et al. [19]. In short, a multiplex assay was used for cfDNA detection focusing on HPV16, HPV33 and human beta-globin (BG) by dPCR. The primers and probes target either the E6 or E7 gene region of each HPV type. BG served as reference gene. dPCRs were performed in duplicate on the QIAcuity Digital PCR System (Qiagen, Hilden, Germany) using the recommended cycling conditions with the QIAcuity Probe PCR Kit and QIAcuity 26k 24-well nanoplates. In total 5 µL of cfDNA was used per reaction. Reactions were performed in duplicate. To call a sample positive both duplicates had to be positive. Fluorescence thresholds for classifying partitions as either positive or negative were set across all wells on each plate, based on the midpoint between positive and negative partitions observed in a positive control.

## Results

### Patient characteristics

**Fig. 1:**
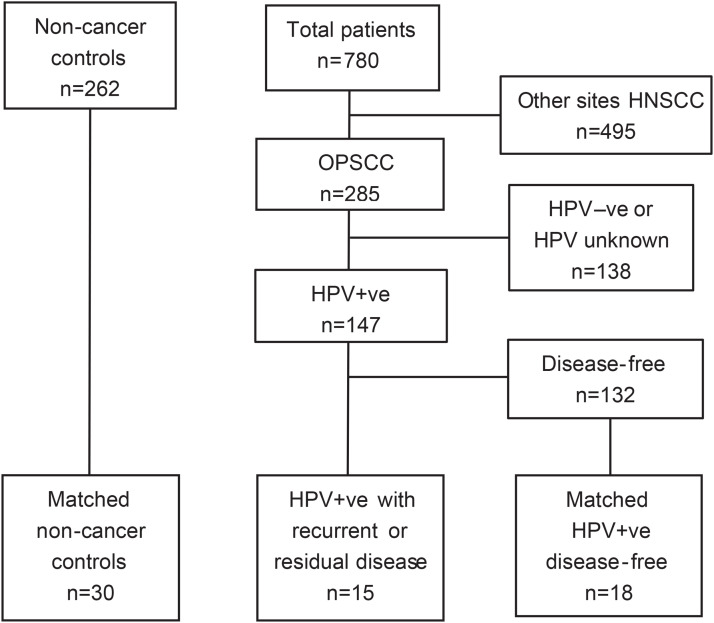
Study design: patients from two studies with plasma biobanking were combined and samples of patients with HPV-positive OPSCC and non-cancer controls (spouses and caregivers), selected for baseline comparisons. From the patient cohort studied longitudinally, six patients showed residual disease, nine recurrent disease, and 18 were disease-free.

Longitudinal plasma and oral rinse samples were collected from 33 patients with HPV-positive OPSCC. The patient group encompassed 6 patients with tumor residue in the neck treated by neck dissection, 9 patients who developed recurrent disease and 18 who remained disease-free for at least 24 months (Fig. 1). In addition, we received the baseline plasma and oral rinse samples from 30 non-cancer controls from the NET-QUBIC biobank. We preselected series with sufficient plasma volumes of 3-4 ml. The majority of patients in the study presented with advanced stage HPV-positive OPSCC tumors, with a large group of never- or minimal smokers. All patients received treatment according to the national and international guidelines. Patient characteristics, including HPV status and treatment are listed in Table 1. The mean plasma DNA yield was 5.3 ng/ml for the OPSCC patients (range 0.15-14.4) and 5.7 ng/ml for the non-cancer controls (range 2.1-21.6), which was not significantly different (*P*=0.9 by Student’s t-test). Median amount of cfDNA was 81% (Details in Supplementary methods).

**Table 1:** Patient and non-cancer control characteristics.

	Recurrent / Residual disease n=15 (%)	Disease free patients in follow-up n=18 (%)	Care givers as non-cancer controls n=30 (%)
Gender			
Male	12 (80)	12 (67)	9 (30)
Female	3 (20)	6 (33)	21 (70)
Age (years)			
Mean	59	63	62
Range	48-68	53-73	37-83
Smoking (pack years)			
Mean	10	13	13
Range	0-39	0-42	0-42
T-stage			
X	1 (7)	2 (11)	
1	5 (33)	3 (16.5)	
2	4 (27)	3 (16.5)	
3	0	0	
4	5 (33)	10 (56)	
N-stage			
0	0	2 (11)	
1	5 (33)	4 (22)	
2	10 (67)	12 (67)	
Primary site			
Unknown Primary	1 (7)	2 (11)	
Oropharynx	14 (93)	16 (89)	
Treatment			
Radiotherapy	15 (100)	18 (100)	
Chemotherapy	8 (53)	3 (17)	
Surgery (+PORT)	6 (40)	12 (67)	

### Comparison of ctHPV-DNA analysis using lcWGS and target-enrichment sequencing

**Fig. 2:**
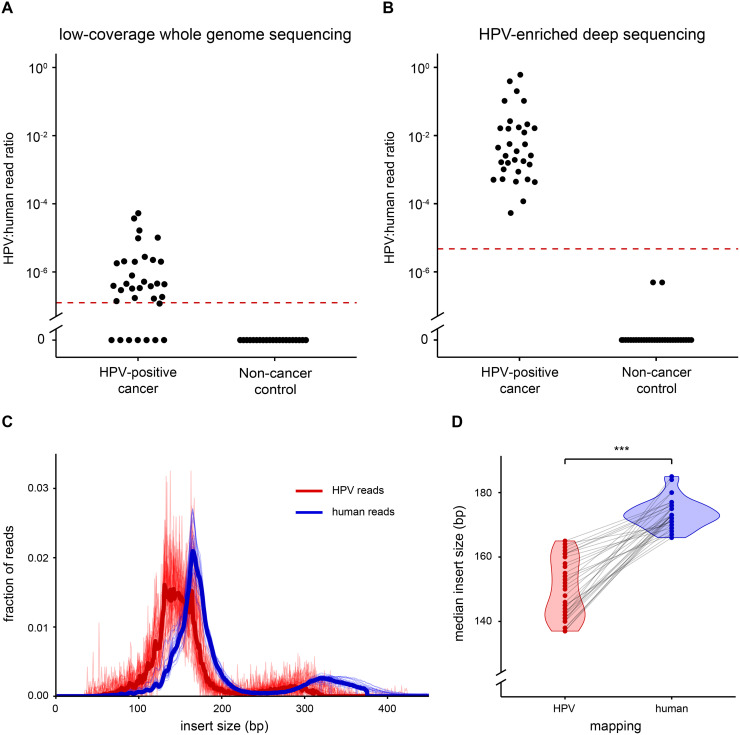
Comparison of ctHPV-DNA sequencing. Samples of OPSCC patients were analyzed by both low coverage whole genome sequencing (lcWGS: left panel A) and target enrichment DNA sequencing (right panel B). On the Y-axis the number of HPV reads per number of human genome reads is depicted. Cut-off levels are indicated with a horizontal bar. In C) the fragment length distributions of HPV (red) and human (blue) genome reads is depicted. In D) the paired median HPV- and human-mapping fragment lengths are compared. The median fragment length of HPV-mapping reads is significantly shorter than that of human-mapping reads (*P*=3.0*10^−14^, paired t-test).

Previously we used low coverage whole genome sequencing (lcWGS) for HPV analysis in plasma [13]. Using this same approach, ctHPV-DNA at baseline was detected in the plasma of 25 out of 33 patients with HPV-positive OPSCCs, and in none of the 21 non-cancer controls (Fig. 2A). This resulted in a sensitivity of 76% and a specificity of 100%. The quality control parameters showed a sufficient concentration of circulating cell-free DNA (cfDNA) for library preparation and sufficient human genome reads in all patients (Supplementary table 2).

To improve the sensitivity of ctHPV-DNA detection, we opted for a target-enrichment step using capture hybridization with probes specific for HPV DNA and only few human genes. The sequencing libraries after target enrichment revealed detection of HPV-derived DNA in plasma of all 33 patients with HPV-positive OPSCCs at baseline (Fig. 2B): with 100% sensitivity a significant improvement over lcWGS (*P*=0.013, McNemar’s Chi-squared test). Absolute mapped HPV16 reads varied from 100 to over a million at baseline in the plasma of OPSCC patients with HPV16-positive tumors (Supplementary table 3). To define statistical cut-offs, we decided to use the number of HPV reads per human genome reads as we also sequenced 29 head and neck cancer genes in the target-enrichment panel. Upon observing the normal distribution of log-transformed read ratios in patients with HPV-positive tumors, we decided on a threshold of the mean log-transformed HPV per human genome read ratio minus three times the standard deviation. This yielded a cut-off read ratio of 4.73 HPV reads per million human genome reads. We note that this cut-off is dependent on the specifics of the composition of the capture panel, i.e. the size of the capture region of HPV and human genome and the relative probe concentrations.

In the 30 non-cancer controls two samples with one HPV16 read were observed and all others had no HPV reads at all, highlighting the perfect separation between HPV16-positive cases and non-cancer controls. Within our cohort we observed 100% sensitivity (95% confidence interval: 0.86–1) when using target enrichment sequencing, in line with the expected ∼99.7% based on the cut-off that was set on basis of 3x the standard deviation. None of the 30 non-cancer control samples reached the cut-off for positivity, in fact most had no HPV reads at all, giving the test a specificity of 100% for detecting HPV-positive samples (95% confidence interval: 0.87–1). For future applications the test accuracies should be determined on additional independent series. To call a ctHPV-DNA negative sample diagnostic, we also used the human genome reads. In theory insufficient coverage of sequencing might cause false-negative calls for ctHPV-DNA. Above 500,000 human genome reads, a sample can be called negative with confidence.

Several lines of evidence indicate that cell-free DNA fragments of tumor cells are smaller than those derived from genomic DNA [20,21]. We therefore analyzed this aspect by comparing HPV DNA fragment lengths with human DNA fragment lengths. In Figs. 2C-D the different size distributions of HPV and human DNA fragments in the plasma are depicted; the median HPV DNA fragment length was 147 bp and that of human DNA 172 bp (*P*=3.0*10^−14^). The fragment sizes of HPV DNA are in line with those reported for tumor cells in other studies. We also explored whether analysis of oral rinses may add to ctHPV-DNA detection using target enrichment sequencing of samples at baseline taken from the same patient and non-cancer control groups. Data are provided in Supplementary table 4. In total, ctHPV-DNA was detected at baseline in 17 of 33 oral rinse samples of patients with an HPV-positive tumor, and we concluded that analysis of oral rinses has no added value.

### Recurrent disease detection by longitudinal ctHPV-DNA analysis

**Fig. 3:**
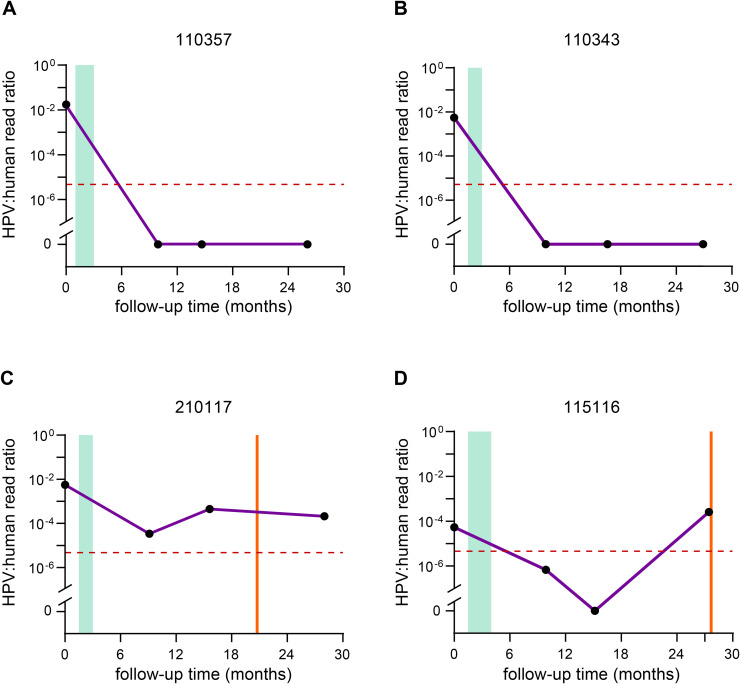
Longitudinal disease monitoring in OPSCC patients. Plasma samples taken at baseline and 6, 12 and 24 months after treatment were collected, and analyzed by target-enrichment sequencing. Plots display the follow-up time in months on the X-axis and HPV/human read ratio on the Y-axis. Green columns represent the treatment period, and a vertical orange line indicates the event: diagnosis of recurrent disease. The horizontal dashed line indicates the cut-off for ctHPV-DNA positivity. A) and B) display two examples of patients who remained disease-free; C) and D) display two examples of patients who developed recurrent disease.

Our next objective was to demonstrate the effectiveness of longitudinal monitoring of ctHPV-DNA in plasma for accurately predicting residual disease or disease recurrence during post-treatment follow-up. Plasma samples of all available time points were analyzed for ctHPV-DNA positivity according to the abovementioned cut-off. Fig. 3 shows representative follow-up plots of two of 18 cases who remained disease-free (Figs. 3A and B). In the 18 disease-free cases analyzed, all 76 samples remained below the threshold except for one single observation; one patient who remained disease-free showed at twelve months one plasma sample just above the background, before it turned to 0 at 24 months.

The plots in Figs. 3C and D demonstrate typical examples of cases developing recurrence. In most cases (7 of 9) ctHPV-DNA remained present above background levels at the earliest time point after treatment, while there were no clinical indications of tumor residue. As an example, a patient presented with a TxN2a OPSCC in the right neck and was treated by a neck dissection right and adjuvant radiotherapy including elective treatment on the contralateral neck and the tonsillar region. In the plasma of this patient, elevated levels of ctHPV-DNA were found at 6 months although not reaching the cut-off, dropped to zero at 12 months and became positive at 24 months. After 27 months a regional recurrence was detected in the left side of the neck, which was treated with a neck dissection and post-operative radiotherapy.

All other cases developing recurrent disease also displayed detectable ctHPV-DNA in the plasma during follow-up and the respective plots are shown in Supplementary Figure S1. The precise timepoints of the samples taken are listed in Supplementary table 6. Hence, above-threshold levels of ctHPV-DNA were detected in the plasma of all 9 recurrent cases. In 7 of 9 cases, ctHPV-DNA was detected in the plasma before imaging or clinical diagnosis of recurrent disease (shown in Figs. 3C,D and Supplementary Figure S1), in some cases a year before clinical diagnosis. In the other cases, the elevated ctHPV-DNA was detected approximately at the same time as the diagnosis of recurrent disease.

### Regional residual disease monitoring by longitudinal ctHPV-DNA analysis

**Fig. 4:**
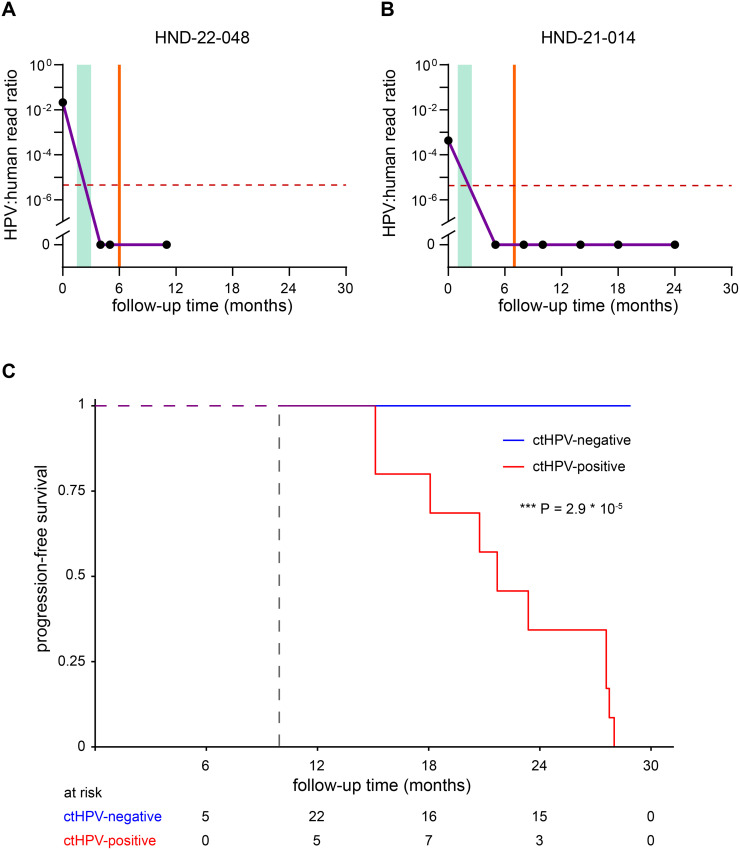
Longitudinal disease monitoring in OPSCC patients with residual disease. Plasma samples taken at baseline and multiple timepoints after treatment were collected and analyzed by target-enrichment sequencing. Plots display the follow-up time in months on the X-axis and HPV:human read ratio on the Y-axis. Green columns represent the treatment period, and a vertical orange line indicates the event: diagnosis of residual disease. The horizontal dashed line indicates the cut-off for ctHPV-DNA positivity. A) and B) display two examples of patients with residual disease in the neck, who were treated by neck dissection. Non-vital tumor was diagnosed by histopathological examination of the surgical specimen. Figure 4C shows disease-free survival of patients of patients stratified by ctHPV-DNA status (as a time-dependent covariate). Time is measured from the first valid measurement after completion of treatment, which was at 6 months for most patients (dashed vertical line). Non-vital tumor residue was not considered an event in this analysis. The number at risk corresponds with ctHPV-DNA status at the specified follow-up time, except at 36 months, when it indicates number of patients still in follow-up. Patients with a ctHPV-DNA positive plasma sample were at significantly higher hazard of recurrence (*P*=2.9*10^−5^, logrank test).

In the studied patient group, six cases were diagnosed with residual disease in the neck (Figs. 4A and B). The physical examination and cytology of patient HND-22-048 was suspect for residual disease, but inconclusive and it was decided to follow an active surveillance, and the patient remained disease-free in line with the ctHPV-DNA sequencing of the plasma that also remained negative during follow-up. Cytology was also not conclusive in the five other cases but these underwent neck dissection. In all but one the pathologist reported a non-vital necrotic lymph node metastasis in the neck (Figs. 4A and B, Supplementary Fig S1). Such non-vital and necrotic metastases can be cleared and have no clinical consequences for the patient when left *in situ*. In four cases, samples were taken after residual disease detection and neck dissection, and these were less informative, although ctHPV-DNA in plasma followed the further recurrence-free disease history. Analysis of blood samples before neck dissection would obviously have been more informative. In the remaining case the absence of ctHPV-DNA in the blood before residual disease detection was in retrospect predictive of the non-vital status after neck dissection.

In Fig. 4C the disease-free survival is depicted of HPV-positive OPSCC patients with and without ctHPV-DNA in plasma during follow-up. The hazard of ctHPV-DNA positive patients was significantly higher (hazard ratio was infinite due to a lack of events in ctHPV-DNA negative patients). Within the ctHPV-DNA positive samples, the levels of ctHPV-DNA were not significantly associated with a higher hazard, although it should be noted that this may be due to the small sample size. On the other hand HPV copy numbers in tumors vary widely.

### Comparison of dPCR and target enrichment DNA sequencing

Two methods are frequently in use at present: dPCR and DNA sequencing. To compare the analytical sensitivity of dPCR and target enrichment sequencing we used a serial dilution of plasma DNA from a HPV-positive OPSCC patient in pooled DNA of a HPV-negative oral cancer patient. The relative lowest limit of detection was determined for the two platforms on split samples of the serial dilution. Both the results of target enrichment sequencing and dPCR are depicted in Supplementary table 5. The target-enrichment DNA sequencing approach shows a 10-100x higher analytical sensitivity than dPCR.

## Discussion

HPV-DNA detection in liquid biopsies has been studied for many years with a variety of methods [11,12,16]. However, there are many hurdles to clinical implementation. Various techniques are being used as well as various read-outs to call a sample positive or negative. For a diagnostic test, standardization is required using generally accepted procedures. Here we demonstrate that diagnostic cut-offs can be determined with robust statistical approaches when applying DNA sequencing. Remarkable is e.g. the large distribution in mapped HPV reads in baseline samples, which might relate to tumor stage, the number of HPV copies in the tumors and other variables, but which may also pose an issue for a diagnostic test.

With our approach, target-enrichment sequencing of HPV16 in plasma demonstrated an accuracy of 100% at baseline. Oral rinse samples will not improve disease detection in HPV-positive OPSCC patients. During longitudinal monitoring, ctHPV-DNA in plasma re-appeared before cancer recurrence. Perhaps even more salient is the finding that ctHPV-DNA negativity accurately reflected the non-vital status of residual tumor mass in the neck after treatment. Measurements of ctHPV-DNA may therefore also be used to prevent unnecessary diagnostic workups for residual disease and prevent overtreatment. Although the numbers are limited, the data presented are convincing, and suggest that liquid biopsy would be a great aid in the clinic, certainly when measurements will be incorporated at every follow-up visit. Also when more samples are analyzed around the moment of response monitoring by imaging, the status of the disease could be well predicted. A further interesting approach would be sampling during treatment such as radiotherapy, which could be used to adapt the treatment.

A challenging question is whether DNA sequencing might be preferred over (droplet) digital PCR. DNA sequencing has a higher analytical sensitivity [15], but whether this is required in the clinic for disease monitoring remains to be determined. But sequencing has other advantages, particularly when human genes are present in the panel in addition to HPV genomes. The data mapped to the human genome provides an opportunity for quantification, and setting robust statistical cut-offs to call samples positive or negative, including considerations on whether a sample was diagnostic or not. When combined with the cfDNA fraction analysis of samples on a Agilent TapeStation using the e Cell-free DNA ScreenTape assay, this approach provides a tremendous opportunity for quality controls in diagnostic applications. Sequencing also allows a check on sample mix-ups through single nucleotide polymorphism comparison. It also offers additional information such as integration sites, fragment lengths, HPV variants, and HPV lineages. In addition, fragmentomics, the use of specific features of the tumor-derived DNA, can be applied to enhance the detection of tumor DNA further. We showed that the HPV reads from the tumor are significantly smaller than the human genome reads that come mostly from normal cells. Although sequencing typically has longer turnaround times than dPCR, it can still be completed in five days in optimal settings. In contrast, dPCR has simpler sample preparation, faster assay times, and a possibly more straightforward data analysis. However, with sequencing costs around €100 per sample, this is not a major obstacle for clinical use.

In multiple studies ddPCR is applied as technique for detection of ctHPV-DNA [22], and generally the results have a promising outlook. In the study of Warlow et al. it was shown that re-appearance of ctHPV-DNA following initial clearance strongly predicted recurrence, with 7 out of 8 patients with rising ctDNA levels developing recurrent disease, while all 40 patients with undetectable ctHPV-DNA remained disease-free. Another study reported five cases in which ctHPV-DNA, initially undetectable post-treatment, rose either before or simultaneously with confirmed disease progression [23]. The NavDx diagnostic platform of Naveris is commercially available based on circulating ctHPV-DNA analyzed by a TTMV® DNA blood test. Three different studies used the circulating TTMV® DNA blood test and showed a sensitivity ranging from 55-90,5%, all with a specificity of 100% [22,[24], [25], [26]]. Based on our comparison, target-enrichment DNA sequencing demonstrates a better performance, showing 10-100x higher analytical sensitivity compared to a regular ddPCR platform. More studies support our observation. The study of Leung et al. compared a sequencing approach for HPV ctDNA detection with dPCR [15]. In a small study of 13 OPSCC patients HPV-seq outperformed dPCR, achieving 100% sensitivity at baseline of OPSCC patients. The recent study of Bryan et al. also demonstrated the highest sensitivity for DNA sequencing over ddPCR when detecting ctHPV-DNA in plasma at baseline [27].

Only few studies have utilized sequencing as a technique for the detection of ctHPV-DNA in HNSCC. The study of Naegele et al. demonstrated the potential of ctHPV-DNA sequencing [28] for detecting HPV-related cancer before clinical diagnosis. Lee et al. found that elevated ctHPV-DNA levels during disease monitoring using DNA sequencing, preceded radiological recurrence by 18 months, even when clinical and PET-CT exams were negative. In contrast, three PET-positive patients with negative biopsies had undetectable ctHPV-DNA, and subsequent imaging confirmed no disease [11]. Larger studies are crucial for understanding the full potential and limitations of sequencing.

HPV positive HNSCC affects primarily younger patients, making long-term treatment-related toxicities a significant concern and research for de-escalation of treatment more important [29]. The study of Chera et al. demonstrates association between ctHPV-DNA and treatment response in HPV positive OPSCC patients, a rapid clearance of ctHPV-DNA followed by definitive CRT was associated with high local control rates, suggesting that per-treatment monitoring could be applied to adapt therapy [30]. O’Boyle et al. conducted a prospective study on ctHPV-DNA clearance after TORS, analyzing 12 patients [31]. They found that persistent ctHPV-DNA on postoperative day one correlated with residual disease, aiding early adjuvant treatment decisions. Patients with macroscopic residual disease had significantly higher ctHPV-DNA levels compared to those without pathological risk factors.

A number of adaptive treatment trials have been initiated. One example is the SIRS 2.0 trial that will stop inclusion February 2026 [32]. Of note, at this moment treatment de-escalation for HPV-related OSCCs can not be recommended outside the framework of clinical trials.

Our study has some limitations. Despite inclusion of 863 HNSCC patients with 285 OPSCC tumors, only 15 patients with recurrent HPV-positive oropharyngeal cancer could be selected. In addition, sampling was performed at baseline, 6, 12, and 24 months after treatment in the NET-QUBIC study, thereby missing data compared to the more frequent sampling schedule at every follow-up visit in the HN-detect study. The latter should become the standard, possibly extended with multiple sampling moments during treatment and around the treatment response imaging.

The results of this study suggest that target-enrichment deep sequencing of HPV DNA (ctHPV-DNAseq) in plasma is a powerful tool for monitoring ctHPV-DNA at least after treatment for HNSCC. The high sensitivity and specificity of our target enrichment sequencing with associated data analysis pipeline and robust statistical cut-offs, permits early detection of recurrence, which could lead to earlier intervention and improved outcomes for patients. Besides the disease monitoring, this assay has several additional potential applications including early diagnosis, screening, and adaptive treatment. Based on the data presented, a complete substitution of the current follow-up policy by liquid biopsy might become a real option.

## Ethics approval and consent to participate

Both studies were approved by the Institutional Review Board, and all participants signed informed consent.

## Availability of data and material

The datasets supporting the conclusions of this article are included within the article and its additional files. All raw data and code will be made available upon request to the corresponding author under an MTA to protect privacy of the patients.

## Funding

This research project was funded by the KWFKankerbestrijding with grant number 12079 and was awarded to prof. dr. R.H. Brakenhoff.

## CRediT authorship contribution statement

**A.S. Pierik:** Writing – original draft, Visualization, Project administration, Investigation, Formal analysis, Data curation, Conceptualization. **J.B. Poell:** Writing – review & editing, Visualization, Validation, Supervision, Software, Methodology, Investigation, Formal analysis, Data curation, Conceptualization. **A. Brink:** Writing – review & editing, Visualization, Software, Resources, Methodology, Investigation, Formal analysis, Data curation. **M. Stigter-van Walsum:** Writing – review & editing, Data curation. **F. Jansen:** Writing – review & editing, Resources. **R. de Bree:** Writing – review & editing, Resources. **J. Hardillo:** Writing – review & editing, Resources. **J.A. Langendijk:** Writing – review & editing, Resources. **R.P. Takes:** Writing – review & editing, Resources. **F. Lamers:** Writing – review & editing, Resources. **I.M. Verdonck-de Leeuw:** Writing – review & editing, Resources. **J.J. Hendrickx:** Writing – review & editing, Resources. **S.A. Koppes:** Writing – review & editing, Resources. **F. Rosing:** Formal analysis, Investigation, Methodology, Writing – review & editing. **T. Waterboer:** Formal analysis, Investigation, Methodology, Resources, Supervision, Writing – review & editing. **C.R. Leemans:** Writing – review & editing, Supervision, Methodology, Funding acquisition, Conceptualization. **R.H. Brakenhoff:** Writing – review & editing, Supervision, Project administration, Methodology, Investigation, Funding acquisition, Conceptualization.

## Declaration of competing interest

None.

## Appendix Supplementary materials

Image, application 1

Image, application 2

Image, application 3

Image, application 4

Image, application 5

Image, application 6

Image, application 7

Image, application 8

Image, application 9

## References

[1] Siegel R.L., Miller K.D., Jemal A. (2019). Cancer statistics, 2019. CA Cancer J. Clin..

[2] Ferlay J., Soerjomataram I., Dikshit R., Eser S., Mathers C., Rebelo M. (2015). Cancer incidence and mortality worldwide: sources, methods and major patterns in GLOBOCAN 2012. Int. J. Cancer.

[3] Leemans C.R., Snijders P.J.F., Brakenhoff R.H. (2018). The molecular landscape of head and neck cancer. Nat. Rev. Cancer.

[4] Chaturvedi A.K., Engels E.A., Pfeiffer R.M., Hernandez B.Y., Xiao W., Kim E. (2011). Human papillomavirus and rising oropharyngeal cancer incidence in the United States. J. Clin. Oncol..

[5] Mahal B.A., Catalano P.J., Haddad R.I., Hanna G.J., Kass J.I., Schoenfeld J.D. (2019). Incidence and demographic burden of HPV-associated oropharyngeal head and neck cancers in the United States. Cancer Epidemiol. Biomark. Prev..

[6] Stein A.P., Saha S., Kraninger J.L., Swick A.D., Yu M., Lambert P.F. (2015). Prevalence of human papillomavirus in oropharyngeal cancer: a systematic review. Cancer J..

[7] Ang K.K., Harris J., Wheeler R., Weber R., Rosenthal D.I., Nguyen-Tan P.F. (2010). Human papillomavirus and survival of patients with oropharyngeal cancer. N. Engl. J. Med..

[8] Gillison M.L., Chaturvedi A.K., Anderson W.F., Fakhry C. (2015). Epidemiology of human papillomavirus-positive head and neck squamous cell carcinoma. J. Clin. Oncol..

[9] Rohde M., Rosenberg T., Pareek M., Nankivell P., Sharma N., Mehanna H. (2020). Definition of locally recurrent head and neck squamous cell carcinoma: a systematic review and proposal for the Odense-Birmingham definition. Eur. Arch. Otorhinolaryngol..

[10] Adilbay D., Lele S., Pang J., Asarkar A., Calligas J., Nathan C.A. (2022). Circulating human papillomavirus DNA in head and neck squamous cell carcinoma: possible applications and future directions. Cancers (Basel).

[11] Lee J.Y., Garcia-Murillas I., Cutts R.J., De Castro D.G., Grove L., Hurley T. (2017). Predicting response to radical (chemo)radiotherapy with circulating HPV DNA in locally advanced head and neck squamous carcinoma. Br. J. Cancer.

[12] Chera B.S., Kumar S., Shen C., Amdur R., Dagan R., Green R. (2020). Plasma circulating tumor HPV DNA for the surveillance of cancer recurrence in HPV-associated oropharyngeal cancer. J. Clin. Oncol..

[13] Mes S.W., Brink A., Sistermans E.A., Straver R., Oudejans C.B.M., Poell J.B. (2020). Comprehensive multiparameter genetic analysis improves circulating tumor DNA detection in head and neck cancer patients. Oral Oncol..

[14] Schmitt M.W., Fox E.J., Prindle M.J., Reid-Bayliss K.S., True L.D., Radich J.P. (2015). Sequencing small genomic targets with high efficiency and extreme accuracy. Nat. Methods.

[15] Leung E., Han K., Zou J., Zhao Z., Zheng Y., Wang T.T. (2021). HPV sequencing facilitates ultrasensitive detection of HPV circulating tumor DNA. Clin. Cancer Res..

[16] Das D., Hirayama S., Aye L., Bryan M.E., Naegele S., Zhao B. (2024). Blood-based screening for HPV-associated cancers. medRxiv..

[17] Smeets S.J., Hesselink A.T., Speel E.J., Haesevoets A., Snijders P.J., Pawlita M. (2007). A novel algorithm for reliable detection of human papillomavirus in paraffin embedded head and neck cancer specimen. Int. J. Cancer.

[18] Rietbergen M.M., Leemans C.R., Bloemena E., Heideman D.A., Braakhuis B.J., Hesselink A.T. (2013). Increasing prevalence rates of HPV attributable oropharyngeal squamous cell carcinomas in the Netherlands as assessed by a validated test algorithm. Int. J. Cancer.

[19] Rosing F., Meier M., Schroeder L., Laban S., Hoffmann T., Kaufmann A. (2024). Quantification of human papillomavirus cell-free DNA from low-volume blood plasma samples by digital PCR *Microbiol*. Spectr.

[20] Mouliere F., Rosenfeld N. (2015). Circulating tumor-derived DNA is shorter than somatic DNA in plasma. Proc. Natl. Acad. Sci. u S. a.

[21] Tivey A., Church M., Rothwell D., Dive C., Cook N. (2022). Circulating tumour DNA - looking beyond the blood. Nat. Rev. Clin. Oncol..

[22] Jones O., Bola S., Winter S.C. (2025). ctDNA to predict treatment response in head and neck squamous cell carcinoma: a systematic review. Laryngoscope.

[23] Campo F., Paolini F., Manciocco V., Moretto S., Pichi B., Moretti C. (2024). Circulating tumor HPV DNA in the management of HPV+ oropharyngeal cancer and its correlation with MRI. Head Neck.

[24] Berger B.M., Hanna G.J., Posner M.R., Genden E.M., Lautersztain J., Naber S.P. (2022). Detection of occult recurrence using circulating tumor tissue modified Viral HPV DNA among patients treated for HPV-driven oropharyngeal carcinoma. Clin. Cancer Res..

[25] Ferrandino R.M., Chen S., Kappauf C., Barlow J., Gold B.S., Berger M.H. (2023). Performance of liquid biopsy for diagnosis and surveillance of human papillomavirus-associated oropharyngeal cancer. JAMa Otolaryngol. Head. Neck. Surg..

[26] Roof S.A., Jabalee J., Rettig E.M., Chennareddy S., Ferrandino R.M., Chen S. (2024). Utility of TTMV-HPV DNA in resolving indeterminate findings during oropharyngeal cancer surveillance. Oral Oncol..

[27] Bryan M.E., Aye L., Das D., Hirayama S., Al-Inaya Y., Mendel J. (2025). Direct comparison of alternative blood-based approaches for early detection and diagnosis of HPV-associated head and neck cancers. Clin. Cancer Res..

[28] Naegele S., Das D., Hirayama S., Shalhout S.Z., Lee H., Richmon J.D. (2024). ctDNA predicts recurrence and survival in stage I and II HPV-associated head and neck cancer patients treated with surgery. medRxiv.

[29] Morgenthaler J., Trommer M., Khor R., Wada M., Bahig H., Garden A.S. (2024). Can we safely de-escalate HPV(+) oropharyngeal cancers? - A review of current practices and novel approaches. Oral Oncol..

[30] Chera B.S., Kumar S., Beaty B.T., Marron D., Jefferys S., Green R. (2019). Rapid clearance profile of plasma circulating tumor HPV Type 16 DNA during chemoradiotherapy correlates with disease control in HPV-associated oropharyngeal cancer. Clin. Cancer Res..

[31] O'Boyle C.J., Siravegna G., Varmeh S., Queenan N., Michel A., Pang K.C.S. (2022). Cell-free human papillomavirus DNA kinetics after surgery for human papillomavirus-associated oropharyngeal cancer. Cancer.

[32] Chai R.L., Ferrandino R.M., Barron C., Donboli K., Roof S.A., Khan M.N. (2022). The Sinai robotic surgery trial in HPV-related oropharyngeal squamous cell carcinoma (SIRS 2.0 trial) - study protocol for a phase II non-randomized non-inferiority trial. Front. Oncol..

