# Supplementary material for: Supersensitive and robust disease monitoring in oropharyngeal cancer patients by circulating tumor HPV-DNA sequencing (ctHPV-DNAseq)
Source: Transl Oncol. 2026 Apr 1;67:102744. doi: 10.1016/j.tranon.2026.102744 (PMC13068874; doi:10.1016/j.tranon.2026.102744)
Supplement: Supplementary file 1 [file mmc1.docx]

**Legend of supplementary Figures and Tables**

**Supplementary Figure 1 Longitudinal disease monitoring in OPSCC patients**

Plasma samples taken at baseline and approximately 6, 12 and 24 months after treatment were collected and analyzed by target-enrichment sequencing. Plots display the follow-up time in months on the X-axis and HPV:human read ratio on the Y-axis. Green columns represent the treatment period, and a vertical orange line indicates the event: diagnosis of either residual or recurrent disease. Vertical orange dashed line indicates extra events such as distant metastasis. The horizontal dashed line indicates the cut-off for ctHPV-DNA positivity. In A), plots of patients who experienced disease recurrence. In B), plots of patients with residual disease consisting of non-vital tumor cells. Note that plots of patients included in Figures 3 and 4 of the main text are not displayed.

**Table S1 Gene list**

Human genes in the target-enrichment panel for sequencing and list of high-risk HPV types of which E7 was included. The genome of HPV16 was included in the panel in its entirety.

**Table S2 Summary of lcWGS data**

Per plasma sample summary of lcWGS data.

**Table S3 Summary of target-enrichment sequencing data of plasma samples**

Per plasma sample summary of target-enrichment sequencing data.

**Table S4 Summary of target-enrichment sequencing data of oral rinses**

Per oral rinse summary of target-enrichment sequencing data.

**Table S5 Comparison of target-enrichment DNA sequencing with ddPCR**

Plasma DNA of two HPV-positive OPSCC patients was serially diluted in pooled plasma DNA of a HPV-negative OPSCC patient. Samples were split in two and analyzed either by ctHPV-DNAseq or ddPCR on a QIAquity platform. On the left DNA sequencing in reads and on the right ddPCR using either E6 or E7 primers and probes with the number of positive droplets indicated. Positive findings have been marked in green.

**Table S6 Timepoints that blood samples were taken per patient**

Time in months of end of treatment, follow-up collection and if applicable clinical detection of recurrent or residual disease after cancer diagnosis.
