# Supplementary figures and images for: Supersensitive and robust disease monitoring in oropharyngeal cancer patients by circulating tumor HPV-DNA sequencing (ctHPV-DNAseq)
Source: Transl Oncol. 2026 Apr 1;67:102744. doi: 10.1016/j.tranon.2026.102744 (PMC13068874; doi:10.1016/j.tranon.2026.102744)

Supplementary Figure 1

A

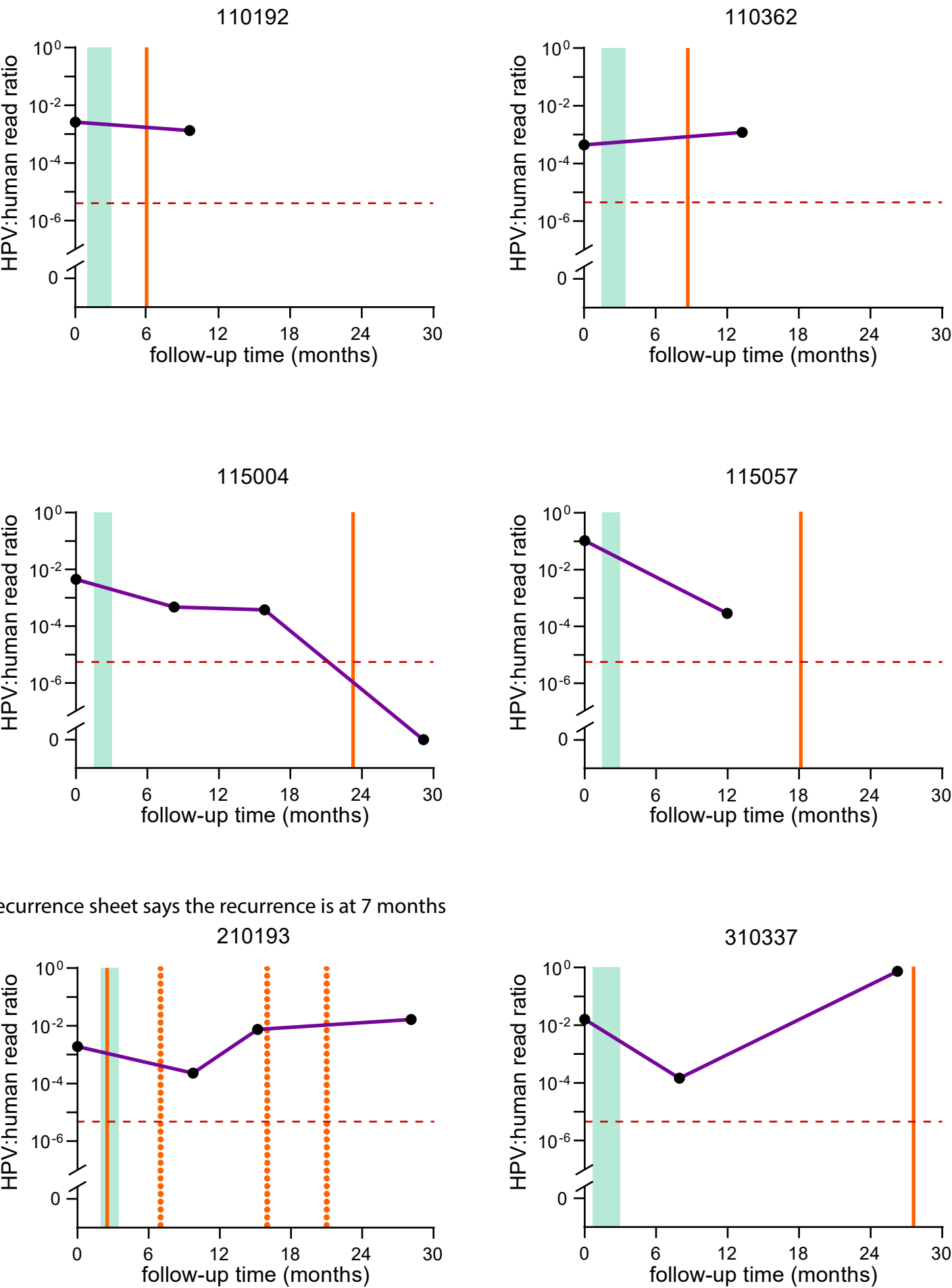

Supplementary Figure 1

A (continued)

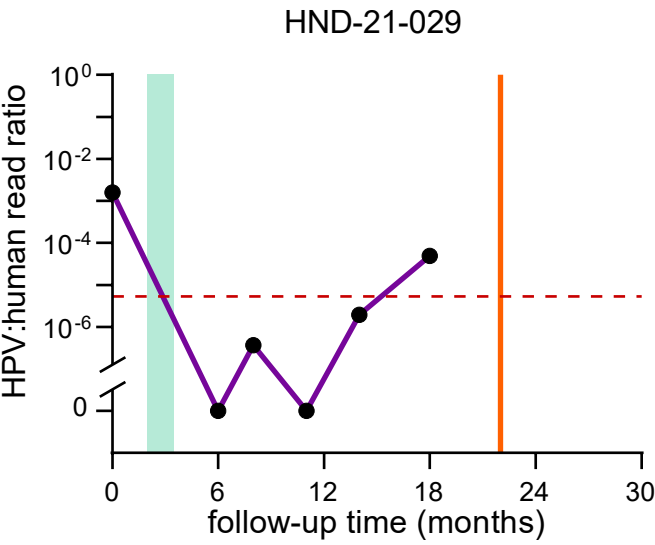

B

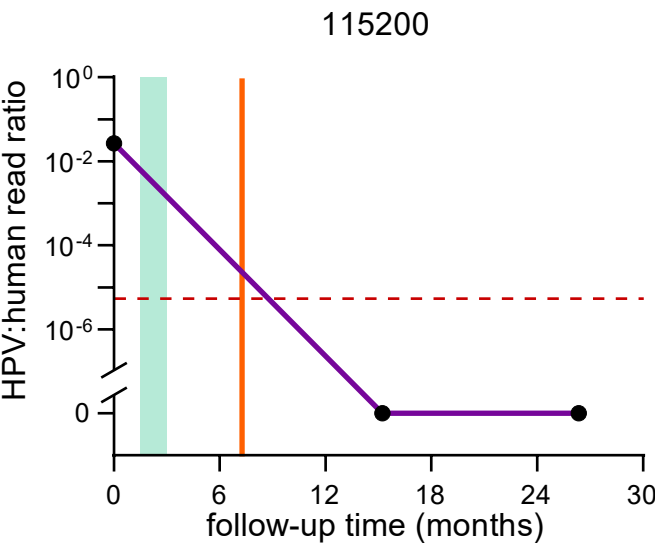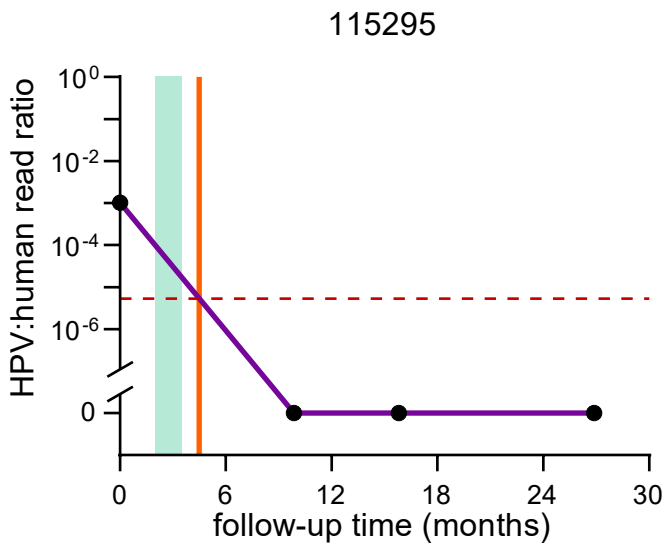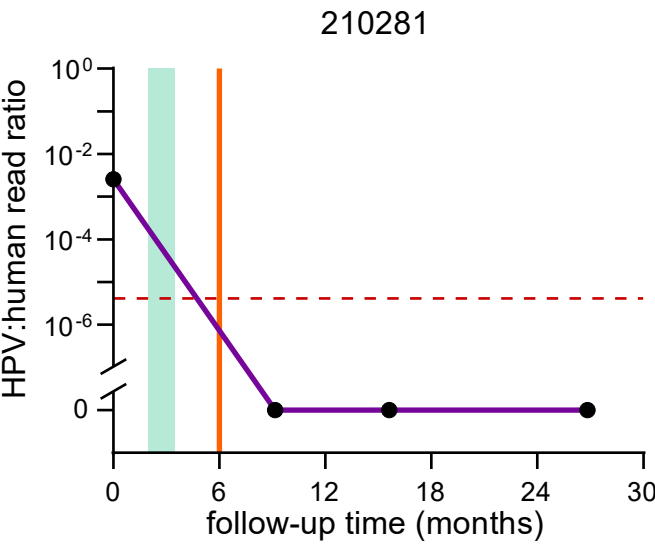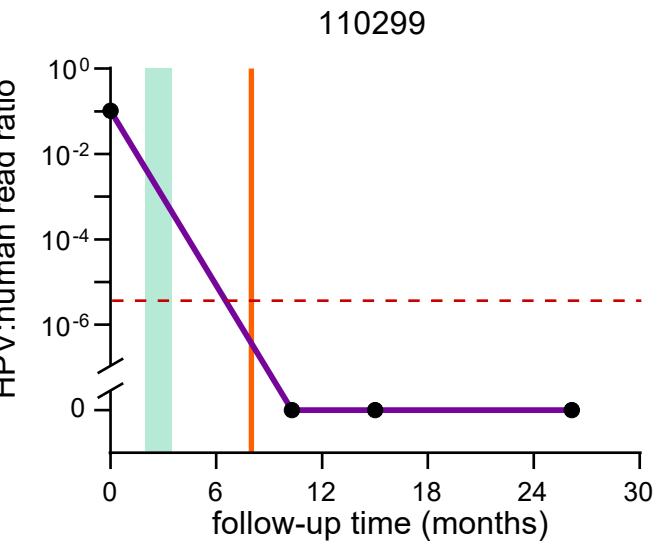

Supplement: Supplementary file 2 [file mmc2.pdf]
