# Supplementary material for: Supersensitive and robust disease monitoring in oropharyngeal cancer patients by circulating tumor HPV-DNA sequencing (ctHPV-DNAseq)
Source: Transl Oncol. 2026 Apr 1;67:102744. doi: 10.1016/j.tranon.2026.102744 (PMC13068874; doi:10.1016/j.tranon.2026.102744)
Supplement: Supplementary file 3 [file mmc3.docx]

**Supplementary methods**

**Blood and oral rinse processing**

Blood was collected in 4 x 6 ml EDTA blood vials and plasma separated by centrifugation at 500g for 20 min. Plasma was transferred to microcentrifuge vials and centrifuged at 21,000g for 10 minutes and pipetted to a new microcentrifuge vial. Alternatively, the blood was centrifuged for 20 minutes at 120g, plasma transferred to a new vial and centrifuged for 360 g for 20 minutes, again transferred and spun for 10 minutes at 21,000g and transferred to a new vial. All centrifugation steps were done at room temperature. Plasma was isolated and frozen within 3 hours after blood draw. In total 3-4 mL plasma was thawed for each available time point. DNA isolation was performed using the QIAamp circulating nucleic acid kit for cell-free DNA (Qiagen) following the protocol of the manufacturer and eluted with 60 µl elution buffer. DNA concentration was measured and quality control performed by loading 2 µl on a cfDNA ScreenTape (Agilent TapeStation). Oral rinse samples were thawed and DNA was isolated using the QIAamp circulating nucleic acid kit for cell-free DNA (Qiagen) following the protocol of the manufacturer and eluted with 60 µl elution buffer.

**DNA isolation blood samples**

We isolated DNA of 3-4 ml plasma, eluted in 60 µl, and loaded 2 µl on a Tapestation cfDNA Screentape to determine the percentage of cfDNA and the concentration of baseline plasma samples. The median percentage of cfDNA was 81%. The mean plasma DNA yield was 5.3 ng/ml for the OPSCC patients (range 0.15-14.4) and 5.7 ng/ml for the caregivers (range 2.1-21.6), these groups were not significantly different (*p*=0.9 by Students T-test).

One example of a Tapestation profile is depicted below.

| 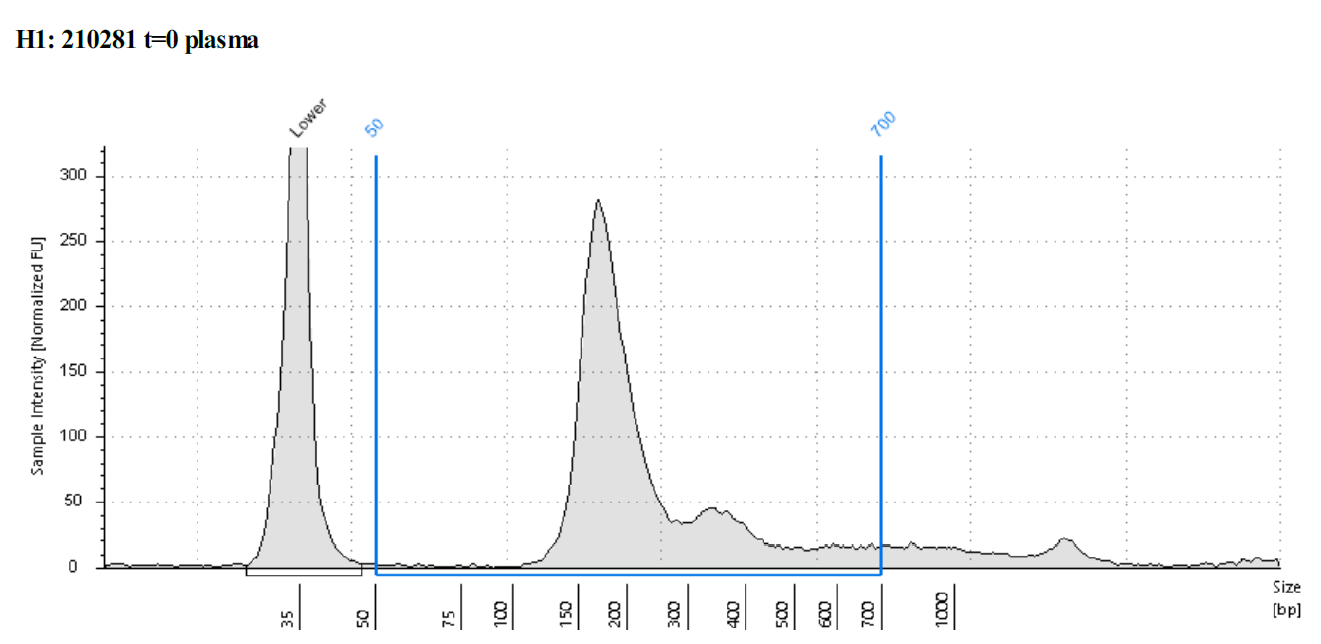 |
| --- |
| 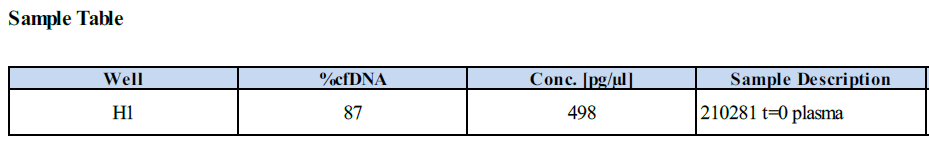 |

| HPV+ve relapse: | %cfDNA | concentration pg/ul |
| --- | --- | --- |
| 110192 | 87 | 275 |
| 110299 | 89 | 298 |
| 110362 | 80 | 151 |
| 115004 | 78 | 251 |
| 115116 | 77 | 368 |
| 115200 | 6 | 17000 |
| 115295 | 73 | 1190 |
| 210117 | 27 | 605 |
| 210193 | 77 | 224 |
| 210281 | 87 | 498 |
| 310337 | 85 | 603 |
| HND-21-014 | 86 | 1310 |
| HND-21-029 | 85 | 898 |
| HND-22-048 | 89 | 453 |
|  |  |  |
| HPV+ve disease free in  follow-up |  |  |
| 110187 | 87 | 146 |
| 110240 | 75 | 636 |
| 110251 | 78 | 111 |
| 110343 | Out of range | 48.8 |
| 110357 | 61 | 181 |
| 110383 | 90 | 377 |
| 115168 | 88 | 410 |
| 115315 | 69 | 67.1 |
| 116037 | 4 | 654 |
| 210200 | 81 | 960 |
| 210348 | Out of range | 11.1 |
| 410511 | 91 | 326 |
| HND-21-013 | 84 | 425 |
| HND-21-040 | 88 | 593 |
|  |  |  |
| Non-cancer controls |  |  |
| 120186 | 91 | 330 |
| 120220 | 91 | 262 |
| 120265 | 10 | 806 |
| 120272 | 89 | 294 |
| 125197 | 87 | 565 |
| 125245 | 85 | 438 |
| 125282 | 75 | 446 |
| 125319 | 78 | 190 |
| 126025 | 57 | 365 |
| 126036 | 79 | 146 |
| 220035 | 83 | 279 |
| 220080 | 85 | 201 |
| 220083 | 90 | 213 |
| 220117 | 81 | 147 |
| 220144 | 76 | 301 |
| 220166 | 53 | 191 |
| 220216 | 80 | 233 |
| 220254 | 92 | 858 |
| 220323 | 78 | 449 |
| 220386 | 89 | 399 |
| 220473 | 81 | 135 |
| 120229 | 75 | 1350 |
| 320105 | 73 | 226 |
| 320118 | 88 | 381 |
| 320270 | 84 | 207 |
| 420340 | 73 | 140 |
| 420587 | 83 | 141 |

**Oral rinses**

Patients rinsed their oral cavity with 10 ml 0.9% sterile saline from a small plastic cup and collected the rinse in the same cup. The rinses were aliquoted in 2ml vials and frozen. For DNA isolation, the vials were thawed and DNA isolation was performed using the QIAamp circulating nucleic acid kit for cell-free DNA following the protocol of the manufacturer, with extra proteinase K during the lysis step and eluted with 60 µl elution buffer. DNA concentration was measured and quality control performed using the Qubit dsDNA High Sensitivity kit (Thermo Fisher Scientific).

**DNA Sequencing**

In total 50 µl of DNA eluate was used for sequencing. The sequencing libraries were generated using the KAPA HyperPrep Kit (KAPA Biosystems) including the use of unique molecular indexes (UMIs) and dual sample indexes, following the KAPA_HyperCap_cfDNA_Workflow_v1.1 of the vendor. For low coverage whole genome sequencing analyses, libraries of 24 samples were equimolarly pooled and sequenced for 150 bp paired-end reads on an Illumina NovaSeq 6000. The generated sequencing libraries were used for target-enrichment using KAPA HyperCap protocol (Roche). The capture panel consisted of 30 human genes (including 29 frequently mutated head and neck cancer genes), the full genome of HP16, and the E7 sequences of all other 14 high risk HPV types (Supplementary table 1)(15, 16). Human: HPV enrichment probes were pooled 2 : 1 in the final panel. Target enrichment was performed as described by the vendor. We aimed for equimolar pooling of 24 samples in a combined amount of 1-2 µg DNA. Enriched libraries were sequenced on an Illumina NovaSeq 6000 for 150 bp paired-end reads.

**Analysis**

Reads were mapped using BWA mem (0.7.17) to the human genome hg19 extended with all 15 high-risk HPV genomes. For low coverage whole genome sequencing (lcWGS) data duplicate reads were removed using GATK (4.3.0.0) MarkDuplicates. For target-enriched data duplicate reads were removed using the UMI information with fgbio (2.1.1) GroupReadsByUmi, CallMolecularConsensusReads and FilterConsensusReads. Unique HPV reads and human genome reads were separately counted using samtools (1.16.1) for lcWGS data. For the target-enriched data on-target HPV and human reads were extracted using bedtools (2.29.2). For lcWGS the number of HPV reads per million human genome reads was taken as readout with a cut-off of 0.125 for positive and negative according to Mes et al.(13).

For target-enrichment sequencing, the absolute number of HPV reads and the number of HPV reads per million human genome reads were calculated as final readout. In addition, variants were called by GATK (4.4.0.0) Mutect2 and VarScan 2 (2.4.4) for SNP analysis. The germline SNPs were correlated to confirm sample identities. Total human genome reads were used to calculate whether a sample was diagnostic. Details of the analytical pipeline are described in the Supplementary methods.

**ctHPV-DNA calling**

The sequencing data contains information on HPV reads, human genome reads and coverage. For the lcWGS assay we calculated the number of HPV reads per million human genome reads and set the cut-off at 0.125 HPV reads per million human genome reads as described previously (13).

For the target-enrichment analysis we calculated and used the number of HPV/human genome reads as second read-out as described in the main text, and determined a clinical cut-off between HPV-positive OPSCC cases and non-cancer controls at baseline. Given the predominant result of 0 HPV reads in controls, the controls were marginally informative for determining a cut-off above which a sample should be considered HPV-positive. As an alternative we determined the mean of the log-transformed HPV mapped reads of all 33 HPV16-positive samples minus 3x the standard deviation as clinical cut-off, which statistically encompasses more than 99.9 % of positive samples. This clinical cut-off value was 4.73 HPV reads per million human genome reads. Obviously this cut-off is specific for this particular 32 gene panel that encompasses 126 kb of the human genome. Values above the cut-off indicate a positive sample and equal to or below the cut-off a negative sample. We did not apply an absolute minimum number of HPV reads. The minimal number of HPV reads we observed at baseline was 100 with almost 2 million human genome reads sequenced.

Finally we can use the calculated cut-off to estimate the number of human genome reads required to call a sample with certain confidence negative. The cut-off of 4.73 per million means one HPV read per 211 416 human genome reads. For a 95% confidence level 633346 and for a 90% confidence level 486803 human genome reads should be sequenced to call a sample without HPV reads negative. Hence, above 500,000 human genome reads and no HPV reads, a sample can be called negative with over 90% confidence.

**Other quality controls and analyses**

Routinely we performed SNP calling and analyzed all germline SNPs in a correlation plot comparison to confirm the identity of the samples. Intrapatient SNP variant allele frequencies always had a correlation coefficent of > 0.893 , while interpatient SNP variant allele frequencies always had a correlation coefficient < 0.799. In addition HPV16 variants were manually checked, and HPV genome coverage profiles examined. All these data were used to corroborate sample identity.

**References**

13. Mes SW, Brink A, Sistermans EA, Straver R, Oudejans CBM, Poell JB, et al. Comprehensive multiparameter genetic analysis improves circulating tumor DNA detection in head and neck cancer patients. (1879-0593 (Electronic)).

14. Schmitt MW, Fox EA-OX, Prindle MJ, Reid-Bayliss KS, True LD, Radich JP, Loeb LA. Sequencing small genomic targets with high efficiency and extreme accuracy. (1548-7105 (Electronic)).

15. Comprehensive genomic characterization of head and neck squamous cell carcinomas. (1476-4687 (Electronic)).
